# The Influence of the Blend Ratio in PA6/PA66/MWCNT Blend Composites on the Electrical and Thermal Properties

**DOI:** 10.3390/polym11010122

**Published:** 2019-01-11

**Authors:** Beate Krause, Lisa Kroschwald, Petra Pötschke

**Affiliations:** Leibniz-Institut für Polymerforschung Dresden e.V., Hohe Str. 6, 01069 Dresden, Germany; krause-beate@ipfdd.de (B.K.); Lisa.Kroschwald@gmx.de (L.K.)

**Keywords:** polyamide, carbon nanotubes, melt-mixed blends, electrical properties, crystallinity

## Abstract

It is known that the percolation threshold of polyamide 6 (PA6)/multiwalled carbon nanotube (MWCNT) composites is higher than that of PA66/MWCNT composites under the same mixing conditions and melt viscosity. A series of blends of PA6 and PA66 containing 1 wt % MWCNTs have been prepared to investigate this phenomenon. At contents up to 20 wt % PA66, the blends were not electrically conductive. The electrical resistivity dropped to 10^9^ Ohm∙cm for PA66/PA6 30/70 blends. The resistivity was 10^5^ Ohm∙cm at higher PA66 contents. Differential scanning calorimetry was used to investigate the thermal behavior of blends. The glass transition temperature was almost constant for all blend compositions, indicating that the amorphous phases are miscible. The MWCNT addition influenced the crystallization of PA66 much more than the PA6 crystallization. A heterogeneous crystallization of the polyamide in PA66/PA6 blends took place, and the MWCNTs were mainly localized in the earlier crystallizing PA66 phase. Thus, the formation of the nanotube network and thus the electrical volume resistivity of the PA6/PA66 blends with 1 wt % MWCNTs is significantly influenced by the crystallization behavior. In PA66/PA6 blends up to 60 wt %, the more expensive PA66 can be replaced by the cheaper PA6 while retaining its electrical properties.

## 1. Introduction

Electrically conductive or antistatic polymer materials may be produced through the addition of conductive multiwalled carbon nanotubes (MWCNTs) to insulating polymers using melt mixing. The electrical conductivity of such nanocomposites depends on the properties of the polymer matrix (notably viscosity, surface tension, and crystallinity), the properties of the MWCNTs (notably aspect ratio, surface functionalization, and dispersability), and the relative loadings of the materials.

Polyamides (PA) are partially crystalline thermoplastic materials that exhibit high dynamic load capacity, high rigidity and hardness, high impact resistance, and high wear-, chemical-, and heat-resistance. The high melting temperature allows them to be used as material for applications at higher temperatures. Conversely, they are susceptible to water absorption. They can be easily chemically modified and show good processability [1]. 

The molecular structures of polyamide 6 (PA6) and polyamide 66 (PA66) are very similar, despite their differing syntheses. The primary difference stems from the mechanism whereby hydrogen bonds are formed. In PA66, any functional group can form a hydrogen bond without molecular deformation. In contrast, PA6 can only form hydrogen bonds if the molecules are arranged in opposite directions in planes or they move against one another. These molecular differences influence the properties of the polyamides, as they result in different total energies of the molecular arrangements. Thus, the lower melting temperature of PA6 is a consequence of its difficulty in forming hydrogen bonds with itself. Conversely, the lower water absorption of PA66 is caused by the ease with which it forms hydrogen bonds with itself. PA66 also exhibits increased temperature resistance, stiffness, and abrasion resistance than does PA6. On the other hand, PA6 is easier to process, distorts less, and is cheaper than PA66.

The amorphous parts of PA6 and PA66 are miscible due to the similarity of their chemical structures [2,3]. Due to the existence of only one single dynamic loss tangent peak for studied PA6/PA66 blends in dynamic mechanical analysis, researchers concluded the miscibility of PA6 and PA66 [4]. Theoretical calculations confirm this result [5].

The most common and stable crystal modification of PA6 is the monoclinic α-modification, which was first described by Brill et al. [6]. It is favored at high crystallization temperatures and low cooling rates from melts or during crystallization from solutions. A high cooling rate leads to the crystal transformation from the α-modification to the metastable, hexagonal γ-modification [7,8]. This can transform back into the α-modification upon melting [9]. The difference in the crystal modifications is that in the monoclinic α-unit cell, an opposite hydrogen-bond arrangement exists, whereas in the hexagonal γ-unit cell, there is a co-axial arrangement. Depending on the type of PA6 and processing, α- and/or γ-modification of crystals were found [4,8]. In the presence of nanotubes, PA6 crystals grow only in α-modification [8]. PA66 can crystallize in the triclinic α- and in the pseudo-hexagonal γ-modification [10]. These modifications were described by Sengupta et al. [11] for PA66 and PA66/MWCNT composites prepared by the combination of solution casting and compression molding. Li et al. [12] reported only the α-modification of the crystal structure of PA66 and PA66/MWCNT composites. Wang et al. [4] described only α-crystals in melt-processed PA66. For PA66/carbon nanotubes (CNT) composites, Li et al. [13] described the decoration of CNT by PA66 lamellar crystals in nanohybride shish-kebab structures. Such a formation of crystalline lamellae has also been observed for PA6/MWCNT composites [14]. For both PA types, a heterogeneous crystallization is described, and it is concluded that the polymer matrix crystallizes at the CNT surface and thus a wrapping of the CNTs with polymer chains takes place. Such polymer layers around the MWCNTs interrupt direct contact between adjacent MWCNTs and impede electron transfer in polyamide/MWCNT composites, reducing electrical conductivity. If distances between neighbored nanotubes are greater than the hopping or tunneling distances of electrons, the transfer of electrons can be disturbed and the formation of conductive pathways ceased.

The crystallization behavior of PA6/PA66 blends was studied by several authors [4,15,16,17]. Li et al. [16] prepared PA6/PA66 blends by in-situ polymerization and found that the PA6 crystallinity decreased if 2–10 wt % PA66 was added. PA66 molecules were found to impede the crystallization of PA6, thus lowering the eventual crystallinity. Wang et al. [4] concluded that PA66 crystallites (for PA6 containing 4 or 12 wt % PA66) served to nucleate the crystallization of PA6. Verma et al. [15] prepared PA66/PA6 blends by melt mixing across the full blend composition range. At PA6 contents of 90–100 wt %, only single melting and crystallization peaks—assigned to the PA6 component—were observed. At higher PA66 contents (30–80 wt % PA6), PA6 and PA66 crystallized and melted separately. If PA66 was the major component (0–20 wt % PA6), single melting and crystallization peaks, corresponding to PA66, were observed. For PA6/PA66 blends prepared by solution mixing (0–100 wt % PA6), distinct melting and crystallization peaks were observed for PA6 and PA66. This was influenced by the second component, which was included at a lesser content [17]. Another study—using pVT data—has shown that cooperative crystallization of PA6 and PA66 and the formation of intra-spherulitic structures in the blend occur, as indicated by the common crystallization and the formation of different spherulites [18].

PA6/MWCNT [8,19,20,21,22,23] and PA66/MWCNT [11,12,19,24,25] composites are widely described in the literature, albeit with a range of preparation methods, materials, and characterization methods. Logakis et al. [21,23] found an electrical percolation threshold of 1.7 vol % MWCNT for composites prepared by masterbatch dilution of PA6/20 wt % masterbatch (Hyperion Catalsis International). Saeed et al. [20] reported an electrical conductivity of 3∙10^−5^ S/cm for PA6/MWCNT composites prepared by in-situ polymerization with 5 wt % CNTs, while PA6/3 wt % MWCNT was not found to be conductive. Pourfayaz et al. [14] prepared PA6/0.5 wt % MWCNT (Nanocyl NC7000^TM^) composites by different solution methods based on the phase-inversion process and reported a surface resistivity of 10^7^ Ohm/square. Melt-mixed PA6/MWCNT (NC7000^TM^) composites resulted in an electrical percolation of approximately 2.5 wt %, as reported by Krause et al. [17]. In comparison, electrical percolation thresholds of around 1 wt % were found for PA66/MWCNT (NC7000^TM^) [19] and even 0.04 wt % for PA66/aerosol-synthesized MWCNT based composites [24,25]. Furthermore, MWCNT addition was reported to increase the crystallization temperature in comparison to unfilled PA6 [8,19,20,22,23,24] or PA66 [11,12,19,25].

Due to their attractive and diverse properties, polymer blends serving as matrices for nanocomposites offer interesting opportunities to optimize the overall property profile. The resulting blend-nanocomposite properties depend on the miscibility of the polymers and the localization of the MWCNTs. Many studies are focused on the preparation of nanocomposite blends with a co-continuous morphology and selective localization of nanotubes in one component or at the interface [26,27,28,29,30,31], since the phenomenon of double percolation can be used in such systems [26,27,28,29,30,32].

In the present study, PA66/PA6 blends with and without MWCNTs were prepared by melt compounding and thoroughly investigated. The finding of lower electrical percolation thresholds for PA66/MWCNT composites than those of PA6/MWCNT composites [19] was the starting point for the development of a blend system taking advantage of the better electrical properties of PA66/MWCNT composites, combined with the benefits of PA6. The characterization of the electrical properties and the crystallization/melting behavior focused on MWCNT enrichment in one of the phases or components, although PA6/PA66 represents a miscible blend. 

## 2. Materials and Methods

Two types of polyamide (PA), namely PA6 (Ultramid^®^ B27E) and PA66 (Ultramid^®^ A27E), both from BASF SE (Ludwigshafen, Germany), were used. For the incorporation of multiwalled carbon nanotubes, two masterbatches containing 15 wt % Nanocyl NC7000^TM^ were used, namely Plasticyl™ PA1503 (PA6) and Plasticyl™ PA1501 (PA66), both provided by Nanocyl, S.A. (Sambreville, Belgium). To determine the electrical percolation threshold, the masterbatch was diluted with pure polyamide of the same kind (Plasticyl™ PA1503 and Ultramid^®^ B27E or Plasticyl™ PA1501 and Ultramid^®^ A27E). The PA66/PA6 blend compositions filled with constant 1 wt % MWCNT were prepared by dilution of Plasticyl™ PA1501 (PA66) with Ultramid^®^ B27E (PA6) and Ultramid^®^ A27E (PA66) to vary the PA66/PA6 ratio in the blend. 

Melt compounding was performed at 280 °C and 150 rpm for 3 min using a conical twin-screw microcompounder (Xplore DSM15, Sittard, The Netherlands) with a volume of 15 cm^3^.

Electrical measurements were performed on 0.5 mm pressed plates (pressing conditions: 320 °C, 2 min, hot press PW40EH, Remshalden, germany). A Keithley electrometer E6517A (Cleveland, OH, USA) was used for these measurements in combination with a Keithley Test Fixture 8009 (for composites with resistivities greater than 10^7^ Ohm·cm, Cleveland, OH, USA) or a 4-point test fixture with gold electrodes with a distance of 16 mm between the source electrodes and 10 mm between the measuring electrodes (for conductive samples). 

The thermal behavior (via differential scanning calorimetry (DSC)) of processed materials was characterized using a Netzsch DSC 204 F1 Phoenix instrument (Selb, Germany). This was performed under a nitrogen atmosphere in a temperature range of 0 °C to 290 °C, with a cooling/heating rate of 10 K/min and a run cycle of first heating-cooling and then heating. The crystallinities of the blend components, α, was calculated using the value of enthalpy of melting for 100% crystalline PA6 or PA66 of 255 J/g and 230 J/g [33], respectively. 

For the calculation of crystallinity, α, of homopolymer composites, the following equation was used:

(1) $$\alpha =\frac{∆H_{m}}{∆H_{0}\cdot \left(1-\varphi \right)}\cdot 100\%$$

where ∆*H_m_* is the enthalpy of melting of the composites, ∆*H_0_* is the enthalpy of melting of a 100% crystalline polyamide, and φ is the filler content (wt %) in the composites.

For the calculation of crystallinities, α_PA6_ and α_PA66_, of PA6 and PA66 in PA66/PA6 blends, the following equations were used:

(2) $$\alpha_{PA6}=\frac{∆H_{m,PA6}}{∆H_{0,PA6}\cdot \left(1-\varphi \right)\cdot \omega_{PA6}}\;\;\;\;\;\;\;\;\;\;\;\;\;\;\;\;\;\;\alpha_{PA66}=\frac{∆H_{m,PA66}}{∆H_{0,PA66}\cdot \left(1-\varphi \right)\cdot \omega_{PA66}}$$

where ∆*H_m,PA6_* and ∆*H_m,PA66_* are the enthalpy of melting of the PA6 or PA66 part in the PA66/PA6 blend (integration range 165–230 °C (PA6) and 230–275 °C (PA66)), ∆*H_0_* is the enthalpy of melting for a 100% crystalline polyamide, φ is the filler content (wt %) in the composites, and *ϖ* is the theoretical mass fraction (wt %) in the blend.

The glass transition temperature *T*_g_ of the blends was determined by the half-step method of the second heating curve with only one inflection point seen at any mixing ratio.

Melt rheological measurements were performed using an ARES oscillation rheometer (Rheometric Scientific Inc., Piscataway, NJ, USA) in frequency sweeps under nitrogen atmosphere at 280 °C within the linear viscoelastic range at 10% strain. The gap between the parallel plates (diameter 25 mm) ranged between 1.5 and 2.0 mm. Sweeps with increasing and decreasing frequency between 0.063 and 100 rad/s were performed, where the downward sweep was always used for interpretation except for PA66. Due to the molecular degradation of PA66 during the measurement time of the frequency sweeps (one sweep 8 min), the first sweep from 0.063 up to 100 rad/s was used for the graphs.

The state of CNT macrodispersion in the composites was studied using light transmission microscopy to analyze the remaining primary agglomerates with sizes >5 μm. Thin sections of 5 μm thickness were prepared perpendicular to the extrusion direction of extruded strands using a Leica RM 2155 microtome (Leica Microsystems GmbH, Wetzlar, Germany). The samples were characterized with a BH2 microscope in transmission mode combined with a DP71 camera (Olympus Deutschland GmbH, Hamburg, Germany). The area ratio, A_A_ = A/A_0_, was determined from the light micrographs by calculating the ratio of the area, A, of the remaining agglomerates to the total area of the micrograph, A_0_, (~0.6 mm^2^), using the software ImageJ Version 1.43 g (Bethesda, MD, USA). For quantification, at least seven cuts were investigated for each sample, and the mean values and the standard deviation between the seven cuts were plotted.

Wide-angle X-ray scattering (WAXS) measurements were obtained with a six-circle diffractometer XRD3003 (GE Sensing & Inspection Technologies, Hürth, Germany) with a wave length of 0.15418 nm (Cu Kα radiation) on the processed samples.

## 3. Results

### 3.1. Thermal Properties of PA6/PA66 Blends

**Figure 1:**
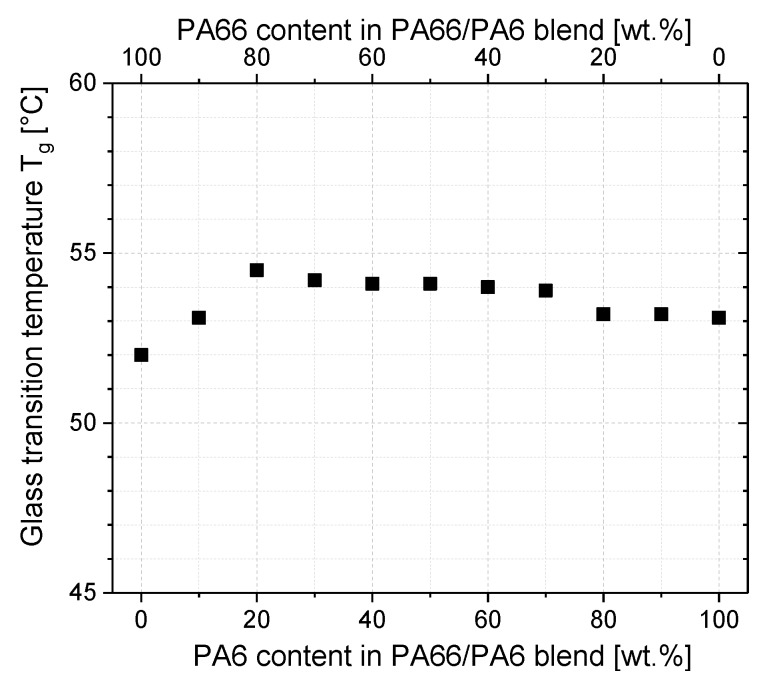
Glass transition temperatures with different PA6/PA66 blend compositions. PA = polyamide.

To evaluate the miscibility of the blends, the glass transition temperatures were determined (Figure 1). With increasing PA6 content, the common glass transition temperature was nearly constant. Thus, it can be concluded that the amorphous phases of PA6 and PA66 are miscible. This finding correlates with the reports by Tomova et al. [2], Ellis et al. [3], and Wang et al. [4] who found that PA6 and PA66 are miscible in the amorphous phase at all blend compositions. 

**Figure 2:**
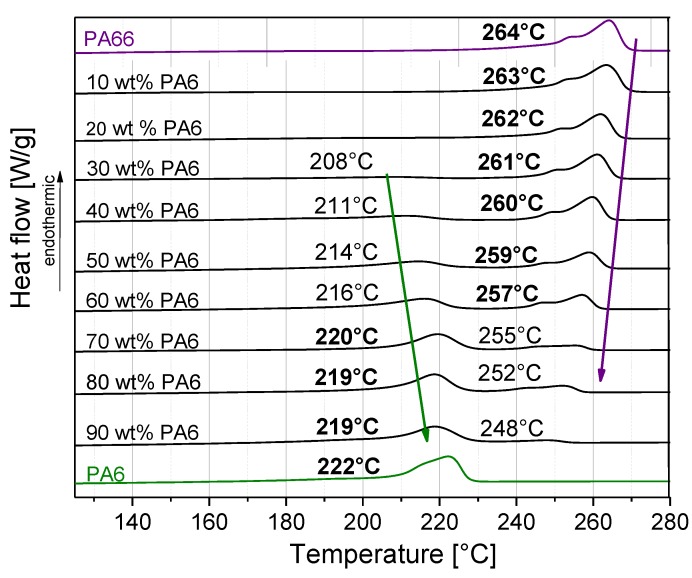
Melting behavior and melting temperatures T_m_ at different PA6/PA66 blend compositions.

The melting behaviors of the PA6/PA66 blends are shown in Figure 2. Heterogeneous melting of the components was found, with one component affecting the melting behavior of the other. The melting temperature of PA66 decreased and the melting temperature of PA6 increased with PA6 content of the blend. At contents equal to or greater than 70 wt % PA6, the melting was dominated by PA6; otherwise PA66 melting behavior was more pronounced, illustrated by the values in bold (Figure 2). The tendency for the melting temperature of PA6 to decrease with increasing PA66 content in PA66/PA6 blends was also reported by Li et al. [16]. The appearance of two melting peaks with increasing PA66 content (4 or 12 wt % PA66 in PA6) was described before by Wang et al. [4], where the peak at the higher temperature was assigned to the melting of PA66.

**Figure 3:**
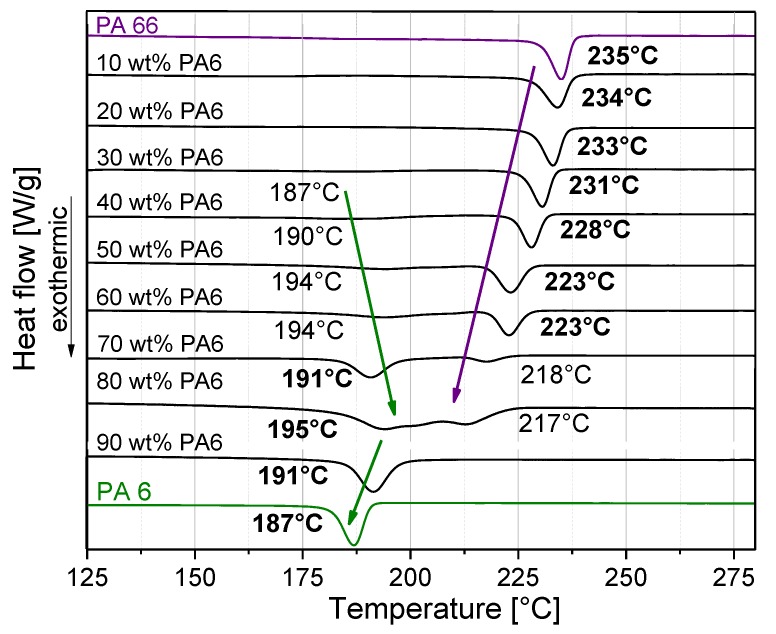
Crystallization behavior and maximum crystallization temperatures *T*_c,max_ at different PA6/PA66 blend compositions.

Heterogeneous behavior was also found in the crystallization of the blends (Figure 3). Similar to the melting behavior, the crystallization behavior of the blends was characterized by a strong mutual influence of the crystallization of the individual components. The crystallization behavior was more pronounced by PA6 at loadings equal to or greater than 70 wt % PA6, while PA66 dominated the crystallization behavior in the remaining blends, as illustrated by the values in bold (Figure 3). Values of onset and maximal crystallization temperature of all blends are summarized in Table 1 and Table 2. For blends with PA6 contents of 0–20 wt %, only one crystallization peak was observed, which can be assigned to the PA66 crystalline phase. It can therefore be concluded that PA6 crystallization was suppressed. Verma et al. [15] has also described such behavior. For blends containing 30–80 wt % PA6, two crystallization temperatures were found. This indicates the separate crystallization of the PA6 and PA66 crystalline phases in the blend. When the blend contained 90 wt % PA6, only a PA6 crystallization peak was visible. With this blend composition, the PA66 crystallization was suppressed. As a tendency, it was observed that both crystallization temperatures (*T*_c,onset_, *T*_c,max_) of PA66 decreased monotonically with the increased PA6 content of the blend. Between 30 and 80 wt % PA6 content, the two crystallization temperatures of PA6 increased, while they decreased at higher PA6 contents.

Li et al. [16] described PA6/PA66 blends containing at most 10 wt % PA66 and found that the PA66 molecules impeded the crystallization of PA6. Wang et al. [4] reported the onset of crystallization as occurring at higher temperatures if PA66 (4 or 12 wt %) was added to PA6, concluding that the promoted PA6 crystallization originates from the nucleation effect of PA66 crystallites. The results of the present study agree well with other studies [15,17].

### 3.2. PA6/PA66/MWCNT Blends

#### 3.2.1. Electrical Properties

**Figure 4:**
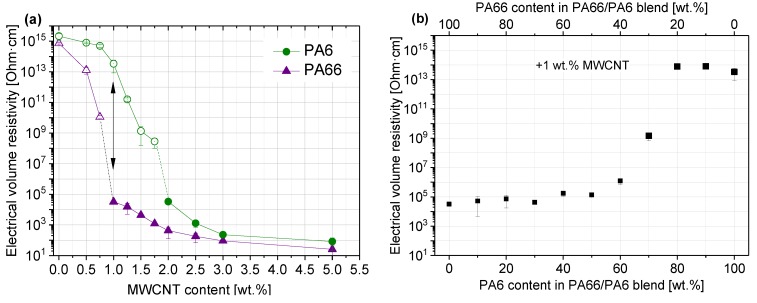
Electrical volume resistivity of (**a**) PA6/MWCNT and PA66/MWCNT composites and (**b**) PA6/PA66 blends filled with 1 wt % MWCNT.

The percolation thresholds for PA6/MWCNT and PA66/MWCNT composites were determined. At the same mixing conditions, the percolation threshold of PA6/MWCNT composites (around 1.5 wt %) was found to be higher than that of PA66/MWCNT composites (0.75–1.0 wt %) (Figure 4a). 

For the preparation of PA6/PA66/MWCNT blends, the MWCNT concentration of 1 wt % was selected as, at this MWCNT concentration, the PA66/MWCNT composite is electrically conductive whereas the PA6/MWCNT composite is not conductive. Due to this difference, the value of electrical resistivity of PA6/PA66/MWCNT blends could be used as a measure for the MWCNT network arrangement. It was found that the blends were electrically non-conductive if the PA6 content exceeded 70 wt % (Figure 4b). At PA6 contents lower than 70 wt %, resistivity values of around 10^5^ Ohm·cm were measured, whereas at 70 wt % PA6, a transition with a resistivity value of 10^9^ Ohm·cm was found.

#### 3.2.2. MWCNT Macrodispersion

**Figure 5:**
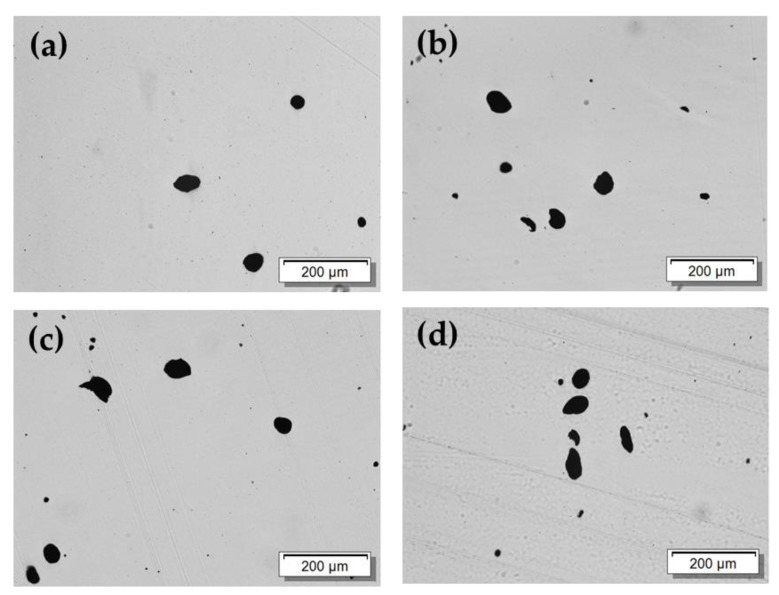
Characteristic light microscopy micrographs of PA66/PA6 blend composites with 1 wt % MWCNT having blend compositions of (**a**) 0/100; (**b**) 40/60; (**c**) 60/40; and (**d**) 100/0 wt %.

**Figure 6:**
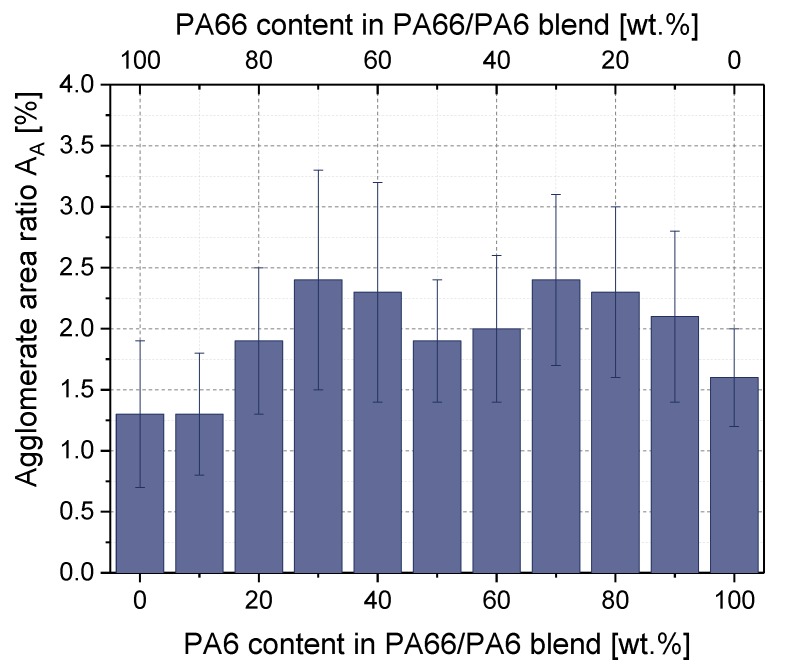
Agglomerate area ratio A_A_ of PA6/PA66/1 wt % MWCNT blends with different blend compositions.

The MWCNT macrodispersion of PA66/PA6 blend composites was studied using transmission light microscopy (LM) on thin sections. As an example, in Figure 5 the LM micrographs of PA66/PA6/1 wt % MWCNT blend composites with different composition are shown. The quantification of MWCNT macrodispersion by calculation of the agglomerate area ratio A_A_ of the composites resulted in mean values between 1.3 and 2.4 %. Considering the high standard deviation, as indicated by the error bars, the CNT macrodispersion in the blends does not differ significantly (Figure 6). In all blend compositions, weak MWCNT macrodispersion with an inhomogeneous distribution of the remaining CNT agglomerates of different sizes was visible. No correlation between the blend composition and the quality of MWCNT macrodispersion could be determined. The best MWCNT macrodispersion was observed for 100 wt % PA66 and PA6/PA66 10/90 composites, as indicated by the lowest A_A_ value of 1.3%.

For selected composites, the MWCNT dispersion in the nanoscale range was additionally observed using transmission electron microscopy (TEM) on thin sections. No significant differences between the composites were found. The composites PA6 as well as PA66 filled with 1 wt % MWCNT and a blend composition PA6/PA66 40/60 + 1 wt % CNT are shown as examples in Figure S1.

#### 3.2.3. Thermal Properties

**Figure 7:**
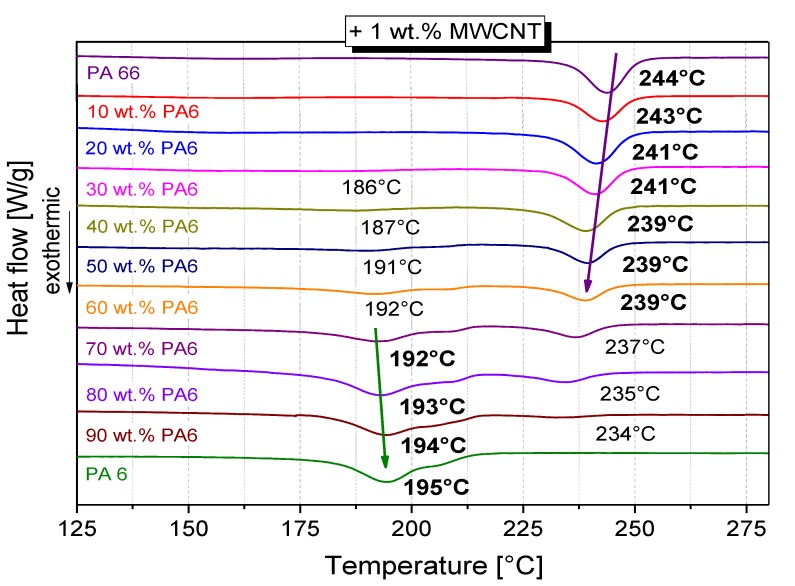
Crystallization behavior of PA6/PA66 blends filled with 1 wt % MWCNT, the values of maximum crystallization temperatures *T*_c,max_ are given on the curves.

The crystallization behavior of MWCNT-filled PA66/PA6 blends was comparable to that of unfilled blends in terms of the occurrence of one or two crystallization peaks (Figure 7). The crystallization temperatures *T*_c,onset_ and *T*_c,max_ for all PA66/PA6 blends as well as PA66 and PA6 composites with and without MWCNTs are summarized in Table 1 (values of PA66 crystallization) and Table 2 (values of PA6 crystallization). Additionally, Table 1 and Table 2 contain the difference of crystallization temperatures (∆*T*_c,onset_, ∆*T*_c,max_) induced by MWCNT addition. Such temperature shifts indicate the nucleation effect of MWCNT.

Regarding PA66 crystallization in blends, the increase in both crystallization temperatures is dependent on the blend composition (Table 1). For PA66/1 wt % MWCNT, the maximum crystallization temperature *T*_c,max_ rose from 235 °C (pure PA66) to 244 °C. With increasing PA6 content in the PA66/PA6 blends, the crystallization temperatures *T*_c,onset_ and *T*_c,max_ of PA66 decreased. However, the difference of the crystallization temperatures between the blends with and without MWCNTs (∆*T*_c,onset_, ∆*T*_c,max_) rose significantly up to 22–23 K (PA66/PA6 20/80). Thus, PA66 crystallization was most clearly influenced by the addition of MWCNT when the blend contained the lowest PA66 content. For the blends with only 10 wt % PA66, PA66 crystallization only occurred when MWCNTs were present.

In contrast, such a dramatic increase in *T*_c,max_ could not be found for the PA6 crystallization (Table 2). Comparing pure PA6 and the PA6/1 wt % MWCNT composite, the crystallization temperature T_c,max_ increased by 7 K from 187 °C to 194 °C, which is comparable to the result of Peoglos et al. [23]. After MWCNT addition in the blends, the maximum crystallization temperature varied only by ± 1–3 K compared to the blend without CNTs. The crystallization temperature *T*_c,onset_ in the blends was also significantly less affected by the addition of MWCNT than in the PA6 composite. With decreasing PA6 content, the ∆*T*_c,onset_ decreased from 22 K (PA66 + 1 wt % MWCNT) to only 8 K (PA66/PA6 40/60 + 1 wt % MWCNT). For blends with a PA6 content of 50% or less, the start of crystallization was hardly influenced by the MWCNT addition. The width of the crystallization peaks increased with increasing PA66 content, which indicates a more incomplete crystallization of PA6 (Figure 7). It can be concluded that the PA66 molecules impede the PA6 crystallization.

The changes in crystallization temperatures show that the crystallization of PA66 was significantly more influenced by the addition of MWCNT than that of PA6. When cooling the melt, PA66 crystallized first and MWCNTs acted as nucleation sites for PA66. Through this, PA66 crystallized at higher temperatures in the presence of MWCNTs. It can be assumed that the PA66 crystals grow on the surface of the nanotubes, as described by [13]. For the subsequent PA6 crystallization at lower temperatures, far fewer uncovered MWCNTs were available, which is why a lower change in crystallization temperature compared to PA66 was observed. It can be concluded that a heterogeneous crystallization of PA66 takes place. This means that the MWCNTs are localized in the vicinity of the crystalline phase of the PA66 in the blend.

In addition, the pronounced widening of the enthalpy peak of PA6 in the blends compared to pure PA6 is an indication of incomplete crystallization, which is even more pronounced in the presence of MWCNTs. For the crystallization of PA66, only a slight widening of the enthalpy peak in the PA66/PA6 blends was observed, which occurred especially with PA66/PA6 20/80 (Table S1). In the blends with MWCNTs, slightly widened peaks were detected for PA66, which became wider with decreasing PA66 content in the blend. However, the peak widening effect was significantly lower in PA66 compared to PA6.

**Figure 8:**
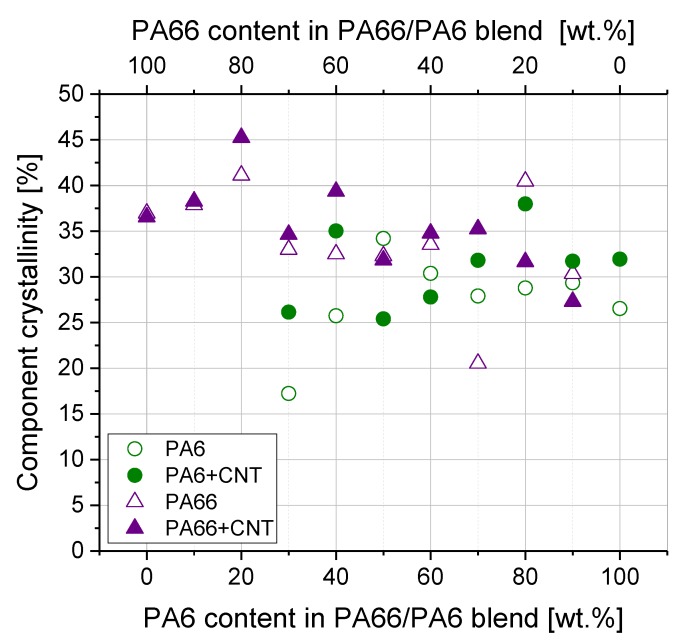
Degree of crystallinity of PA6 and PA66 components in blends without and with 1 wt % MWCNTs. The melting enthalpies attributed to PA6 and PA66 (Table S1) are related to the corresponding content in the blend and the value of 100% crystallinity of that component (calculated using Equation (2)).

To investigate the mutual influence of both crystallization processes, the crystallinity of the individual components in the blends was calculated (Figure 8). The melting enthalpies on which the calculations of crystallinities were based can be found in Table S1. The crystallinity of the components in the blend varied greatly with the blend composition. Sometimes there were large differences between the blends with and without CNTs. The degree of crystallinity of PA6 varied between 25 and 37% when MWCNTs were present and there was no clear general tendency depending on the blend composition. No crystallinity of PA6 was observed at contents below 30 wt % PA6 because in these blends only melting and crystallization of the PA66 component took place. For blends containing 10 or 20 wt % PA6, the PA6 crystallization was completely suppressed. With the addition of MWCNT, PA66 crystallinity increased slightly for most compositions. The degree of crystallinity of PA66 varied between 27 and 44% for blends with MWCNT. In blends with high PA66 contents, the crystallinity in blends with MWCNTs was higher or similar to blends without MWCNTs. At lower PA66 concentrations (20 wt % and lower) the crystallinity decreased. Apart from the fluctuations of the values, the PA66 crystallinity seemed to decrease slightly with increasing PA6 content in the blend and at the same time the PA6 crystallinity increased only very slightly. From these values for crystallinity it can be concluded that there is a cross-influencing of crystallization of PA6 and PA66, independent on the MWCNT presence.

The melting behavior of blends containing CNTs was similar to that of the unfilled blends. The melt temperatures differed only marginally. The DSC curves show two melting peaks for blends containing 30–90% PA6. Otherwise, the peak of the major PA66 component is visible. The DSC melting curves are shown in Figure S2. 

Additionally, the thermal behaviors of the PA66 and PA6 masterbatches with 15 wt % MWCNTs were compared with the base PA66 and PA6 materials and the composites with 1 wt % MWCNTs obtained by masterbatch dilution (Figures S3–S6). No influence of the MWCNT addition on the melting temperature of both polyamides was found, while the peak width was found to increase with increasing MWCNT content (Figures S3 and S4). As shown, MWCNT incorporation lead to an increase of crystallization temperatures, in addition to wider and lower enthalpy peaks with increasing MWCNT content (Figures S5 and S6). Such effects were more pronounced for PA6/MWCNT (Figure S6) than for PA66/MWCNT composites (Figure S5).

**Figure 9:**
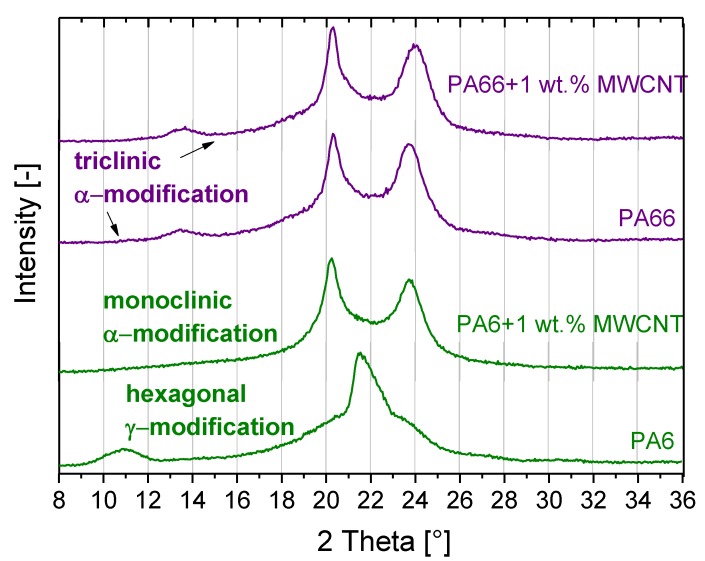
Diffractogram of PA6, PA66, and PA composites containing 1 wt % MWCNT.

The crystalline structure of processed PA6, PA66, and their composites containing 1 wt % MWCNT was investigated using WAXD (Figure 9). For pure PA66, 2 peaks at 20° and 24° could be observed, representing the (100) and (010,110) reflex for the triclinic α-modification of PA66 crystals. The small reflection at 13.6° indicates the presence of relatively few γ-crystals. The incorporation of 1 wt % MWCNTs to PA66 does not change the crystalline structure. However, the (010,110) reflex was found at a slightly higher 2θ angle, indicating a decrease in the size of the elementary cell due to the addition of MWCNTs. Li et al. [12] found only the triclinic α form of crystallites, using WAXD, for PA66/MWCNT composites. The presence of α-modification and comparatively little γ-modification of PA66 crystals was described by Sengupta et al. [11] for PA66 and PA66/MWCNT composites. 

For PA6, a significant peak at 22° ((100) reflex) and a small peak at 11° ((002) reflex) were detected, showing the presence of a hexagonal γ-cell, which is similar to findings on melt-mixed PA6 studied by Wang et al. [4]. PA6 may contain low concentrations of monoclinic α-modification PA6 crystals, which is suggested by the widening of the peak at 22°. In contrast to the unfilled PA6, the PA6/MWCNT composite crystallized in the stable monoclinic α-modification, as indicated by the (200)- and (002,200) reflexes at 20° and 24°. These findings are in accordance with those described by Phang et al. [8], who found α- and γ-crystals in PA6 and only α-crystals in PA6/MWCNT. 

## 4. Discussion

The aim of this study was to investigate the influence of blend composition on the electrical properties and the crystallization and melting behavior of melt-mixed PA66/PA6/MWCNT blend composites containing 1 wt % MWCNTs. 

The electrical volume resistivity of the PA6 and PA66 composites (as shown in Figure 4) shows that the PA66 composite was electrically conductive at a MWCNT content of 1 wt % (volume resistivity of 3.2 × 10^4^ Ohm·cm), while the PA6 composite was non-conductive (volume resistivity of 4.2 × 10^13^ Ohm·cm). Volume resistivity values of around 10^5^ Ohm·cm were found for blends containing 0–60 wt % PA6 (or 100–40 wt % PA66). These resistivity values were in the same range as that of the PA66/1 wt % MWCNT composite. In contrast, the blends containing 80–100 wt % PA6 (or 20–0 wt % PA66) were not electrically conductive. Based on this stepwise change in the volume resistivity values of the blends at 70 wt % PA6, a non-homogeneous localization of MWCNTs can be assumed. Thereby, the macrodispersion did not differ much between the composites with 1 wt % MWCNTs in PA6, PA66, or at the different blend compositions investigated by light microscopy (Figure 5 and Figure 6). No significant differences can be observed for the MWCNT nanodispersion, which was investigated by transmission electron microscopy (Figure S1).

As another potential influencing factor, the matrix viscosity was considered. As discussed in the literature, lower matrix viscosity helps in the infiltration step of primary nanotube agglomerates and in the retention of nanotube length, simultaneously resulting in improved dispersion and reduced resistivity values [34]. Conversely, in other composite systems it was found that an increased melt viscosity, which leads to higher shear stresses during dispersion, is dominant for improved dispersion and thus reduced electrical resistance [35,36]. As illustrated in Figure S7, PA66 and its composite with 1 wt % MWCNTs have only slightly higher melt viscosities than those of PA6 and PA6/1 wt %. Thereby, the viscosity of MWCNT-filled composites is slightly higher than that of the unfilled polyamides (see Figure S7). The viscosity curves of PA66/PA6/1 wt % MWCNT blend composites with different blend ratios are very similar to the curves of the PA66/1 wt % composite (Figure S8). Based on these very similar viscosity values, an influence of the matrix viscosity on the electrical resistivity or MWCNT dispersion can be excluded.

Therefore, the reason for the difference in electrical resistivity values was assigned to the different crystallization behavior of the blend composites at different blend ratios, which influences the formation of nanotube networks at the nanoscale. With regard to melting and crystallization behavior, the two polyamides influence each other, which applies both to the blends with and without MWCNTs. For blends with MWCNTs, the crystallization of the PA66 component is significantly more influenced than the PA6 fraction. With the addition of MWCNT, the crystallization temperatures of the PA66 component increase illustrating the strong nucleating effect of MWCNTs. Interestingly, the difference in crystallization temperatures for blends with and without MWCNTs increases as the PA66 content decreases (Table 1). No significant shift of the maximum crystallization temperature is observed for the crystallization of PA6. There is only an increase in *T*_c,onset_, which indicates a significant widening of the crystallization peak (Table 2, Figure 7). With regard to the crystallization process, PA66 crystallites are first formed by cooling the miscible melt of PA6/PA66 blends. Due to the formation of polymer layers on the surface of MWCNTs, which is described for polyamides [13,14], it can be concluded that the majority of the CNTs are localized in the vicinity of the PA66 component. With increasing PA6 content, less PA66 is available for crystallization and more MWCNTs are available to affect PA6 crystallization. When the crystallization of PA6 starts, the crystallization process seems to be different to that of PA66. For blends with PA66 as the major component (50–100 wt % PA66), the formation of the electrically conductive network does not seem to be hindered by PA6 crystallization, since the electrical resistivity is composition-independent (Figure 4b). A slight increase in volume resistivity can be observed for the blend PA66/PA6 40 wt %/60 wt %. Starting at 70 wt % PA6 in the blend composition, a significant increase in resistivity is measured and it is concluded that the majority of MWCNTs are localized in the PA6 component. As described by Brosse et al. [14], a trans-crystalline phase is formed around the carbon nanotubes during the PA6 crystallization, and the formation of conductive pathways is impeded. Another reason could be the hindrance of PA6 crystallization by PA66, as described by Li et al. [16]. In good agreement with [16], a decrease of the melting enthalpy ∆*H_m_* of PA6 with increasing PA66 content was also observed in the present study (Table S1), and also the melting enthalpy ∆*H_m_* of PA66 decreased in the blends. For both polyamides, the component crystallinity decreases slightly with decreasing proportion (Figure 8). This is a further indication that the PA6 and PA66 molecules affect each other in their crystallization. A significant widening of the crystallization peaks in the blends can be interpreted as an indication of incomplete crystallization, which is more pronounced for PA6 than for PA66.

## 5. Conclusions

The electrical volume resistivity of the PA6/PA66 blends with 1 wt % MWCNTs was found to be significantly influenced by the blend composition, with conductivity in PA66-rich blend composites and non-conductivity in PA6-rich blend composites. The crystallization behavior of the blends and the differences between PA66 and PA6 were identified as the main influencing factors. Based on a literature report [14], it was considered that a trans-crystalline PA6 crystalline layer, that covers the carbon nanotubes and hinders the formation of the electrical percolation network of the MWCNTs, is formed during the crystallization of PA6. The extent of this nanotube covering depends on the blend ratio. If the PA66/PA6 blends contain 40 wt % or more PA66, the blends were electrically conductive. This is expected to be due to the localization of MWCNTs mainly in the vicinity of the PA66 component. While maintaining the electrical performance of the blend, up to 60 wt % of the more expensive PA66 can be replaced by the less expensive PA6.

## Figures and Tables

**Table 1:** Crystallization temperatures *T*_c, onset_ and *T*_c, max_ of PA66/PA6 blends filled with 0 or 1 wt % multiwalled carbon nanotube (MWCNT) and their differences (∆*T*_c, onset_, ∆*T*_c, max_) regarding PA66 crystallization.

Blend Composition	*T*_c, onset_ (°C) PA66			*T*_c, max_ (°C) PA66		
	@ 0 wt % CNT	@ 1 wt % CNT	∆*T*_c, onset_ (°C)	@ 0 wt % CNT	@ 1 wt % CNT	∆*T*_c, max_ (°C)
PA66	238.0	250.9	12.9	235.0	244.1	9.1
PA66/PA6 90/10	237.8	250.0	12.2	234.1	243.0	8.9
PA66/PA6 80/20	236.3	249.0	12.7	233.0	241.4	8.4
PA66/PA6 70/30	233.7	248.2	14.5	230.6	241.1	10.5
PA66/PA6 60/40	231.4	246.5	15.1	228.1	239.1	11.0
PA66/PA6 50/50	227.9	246.3	18.4	223.3	239.5	16.2
PA66/PA6 40/60	227.3	246.0	18.7	222.8	239.1	16.3
PA66/PA6 30/70	223.4	244.3	20.9	217.8	236.8	19.0
PA66/PA6 20/80	220.6	242.9	22.3	211.4	234.5	23.1
PA66/PA6 10/90	-	242.4	-	-	234.3	-
PA6	-	-	-	-	-	-

**Table 2:** Crystallization temperatures *T*_c, onset_ and *T*_c,max_ of PA66/PA6 blends filled with 0 or 1 wt % MWCNT and their differences (∆*T*_c, onset_, ∆*T*_c, max_) regarding PA6 crystallization.

Blend Composition	*T*_c, onset_ (°C) PA6			T_c, max_ (°C) PA6		
	@ 0 wt % CNT	@ 1 wt % CNT	∆T_c, onset_ (°C)	@ 0 wt % CNT	@ 1 wt % CNT	∆T_c, max_ (°C)
PA66	-	-	-	-	-	-
PA66/PA6 90/10	-	-	-	-	-	-
PA66/PA6 80/20	-	-	-	-	-	-
PA66/PA6 70/30	200.8	196.3	−4.5	186.7	185.7	−1.0
PA66/PA6 60/40	202.8	199.9	−2.9	189.5	187.4	−2.1
PA66/PA6 50/50	201.5	202.8	1.3	193.7	190.7	−3.0
PA66/PA6 40/60	204.1	212.4	8.3	193.7	191.7	−2.0
PA66/PA6 30/70	194.7	213.5	18.8	190.8	192.1	1.3
PA66/PA6 20/80	202.4	214.2	11.8	194.8	193.1	−1.7
PA66/PA6 10/90	197.2	218.3	21.1	191.4	194.3	2.9
PA6	190.7	212.6	21.9	186.9	194.7	7.8

## Supplementary Materials

The following is available online at http://www.mdpi.com/2073-4360/11/1/122/s1, Figure S1. TEM micrograph of (a) PA6+1 wt % MWCNT, (b) PA66+ 1 wt % MWCNT, (c) PA66/PA6 40/60 +1 wt % MWCNT (thin section, Zeiss Libra 120); Figure S2. Melting behavior of PA66/PA6/1 wt % MWCNT composite including melting temperature *T*_m_ (2^nd^ heating); Figure S3. Melting behavior of PA66, PA66/1 wt % MWCNT (masterbatch dilution), and PA66/15 wt % MWCNT (masterbatch Plasticyl^TM^ PA1501) (2^nd^ heating); Figure S4. Melting behavior of PA6, PA6/1 wt % MWCNT (masterbatch dilution), and PA6/15 wt % MWCNT (masterbatch PlasticylTM PA1503) (2nd heating); Figure S5. Crystallization behavior of PA66, PA66/1 wt % MWCNT (masterbatch dilution), and PA66/15 wt % MWCNT (masterbatch Plasticyl^TM^ PA1501); Figure S6. Crystallization behavior of PA6, PA6/1 wt % MWCNT (masterbatch dilution), and PA6/15 wt % MWCNT (masterbatch Plasticyl^TM^ PA1503); Figure S7. Complex viscosity of PA6 and PA66 and their composites filled with 1 wt % MWCNT; Figure S8. Complex viscosity of PA66/PA6/1 wt % MWCNT blends; Table S1. Melting temperatures ∆H_m_ of PA6 and PA66 in PA66/PA6 blends filled with 0 or 1 wt % MWCNT.
