# Supplementary material for: The Influence of the Blend Ratio in PA6/PA66/MWCNT Blend Composites on the Electrical and Thermal Properties
Source: Polymers (Basel). 2019 Jan 11;11(1):122. doi: 10.3390/polym11010122 (PMC6401933; doi:10.3390/polym11010122)
Supplement: Supplementary file 1 [file polymers-11-00122-s001.pdf]

polymers

MDPI

Article

The Influence of the Blend Ratio in PA6/PA66/MWCNT Blend Composites on the Electrical and Thermal Properties

Beate Krause, Lisa Kroschwald and Petra Pötschke

Supplementary Materials

Transmission electron microscopy (TEM) on PA6/PA66/MWCNT blends

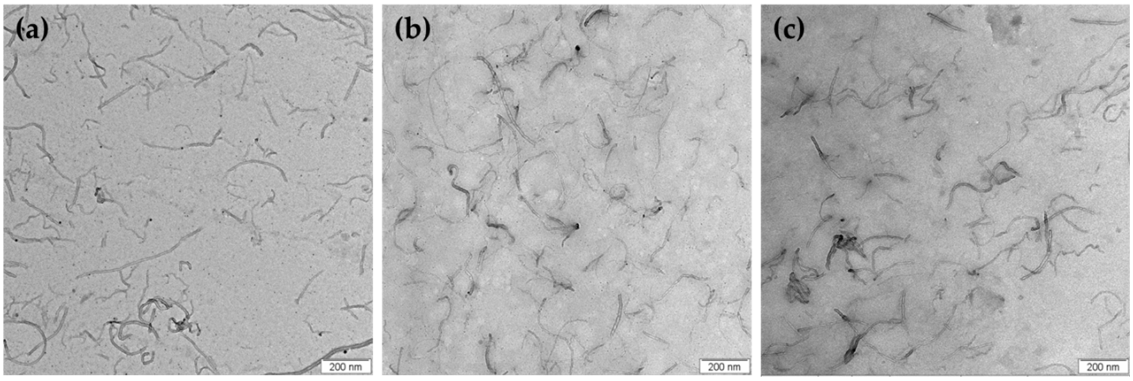

Figure S1. TEM micrograph of (**a**) PA6 +1 wt % MWCNT, (**b**) PA66 + 1 wt % MWCNT, and (**c**) PA66/PA6 40/60 +1 wt % MWCNT (thin section, Zeiss Libra 120).

Differential scanning calorimetry (DSC)

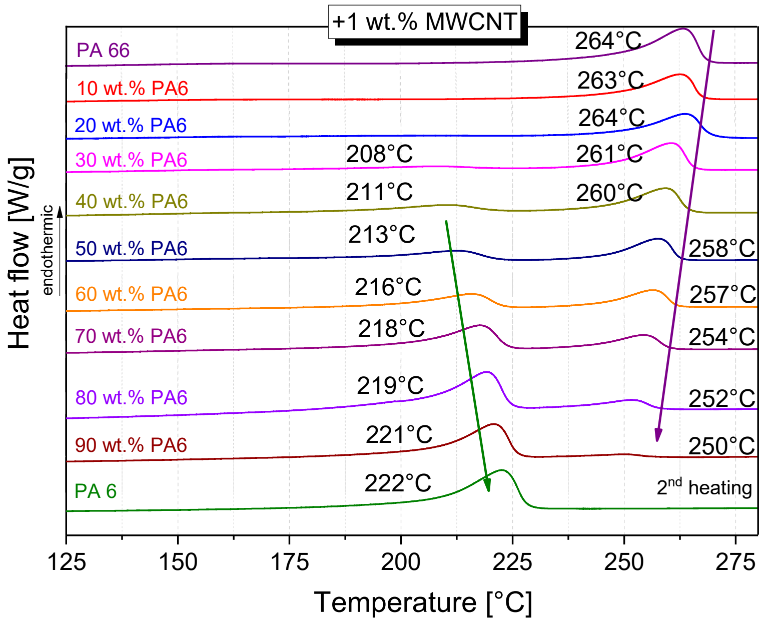

Figure S2. Melting behavior of PA66/PA6/1 wt % MWCNT composite including melting temperature T_m (2nd heating).

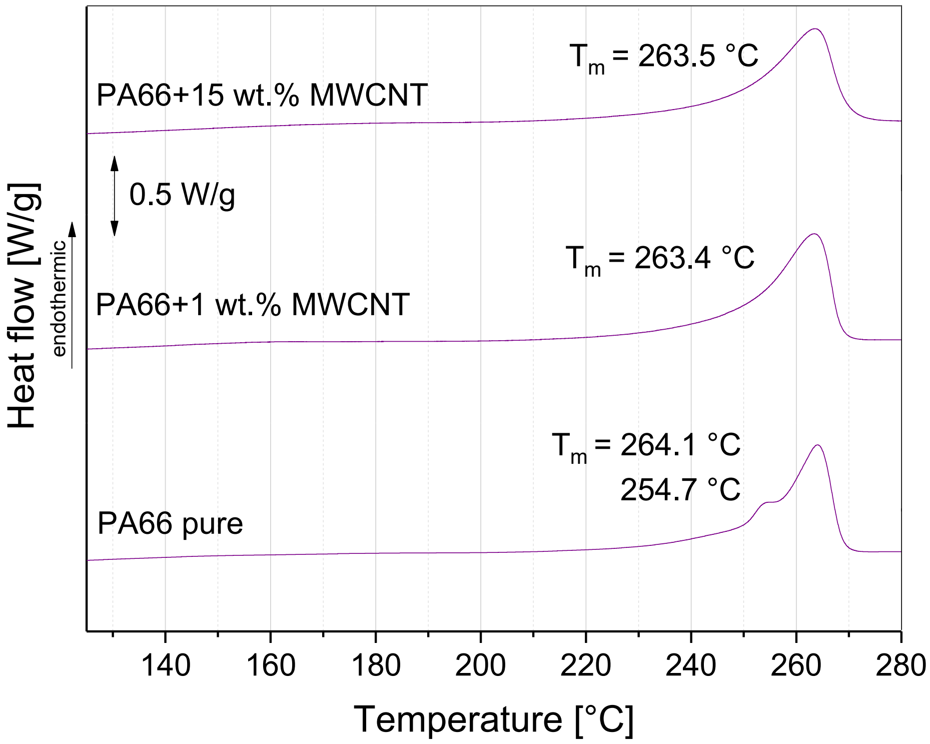

Figure S3. Melting behavior of PA66, PA66/1 wt % MWCNT (masterbatch dilution), and PA66/15 wt % MWCNT (masterbatch Plasticyl™ PA1501) (2nd heating).

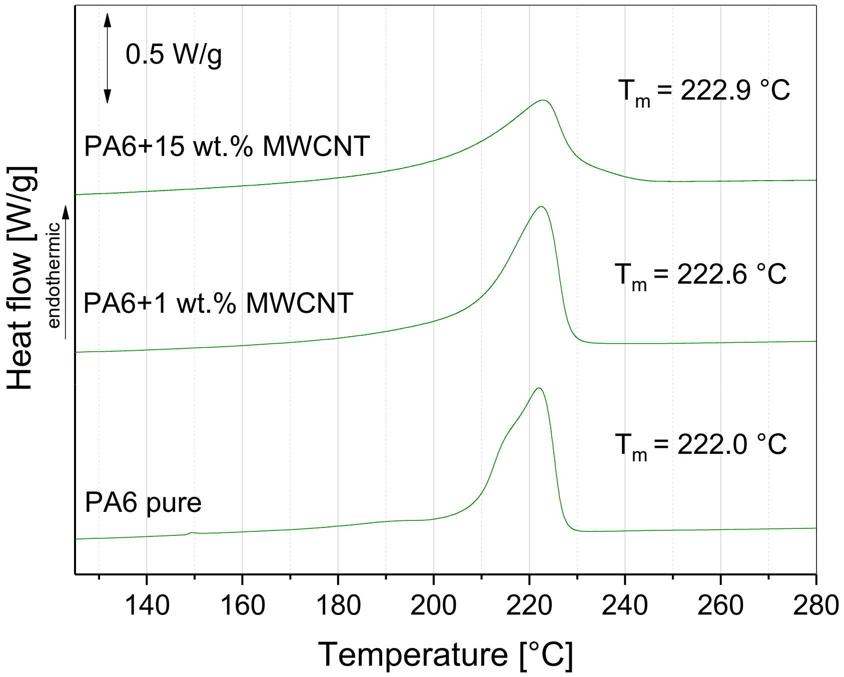

Figure S4. Melting behavior of PA6, PA6/1 wt % MWCNT (masterbatch dilution), and PA6/15 wt % MWCNT (masterbatch Plasticyl™ PA1503) (2nd heating).

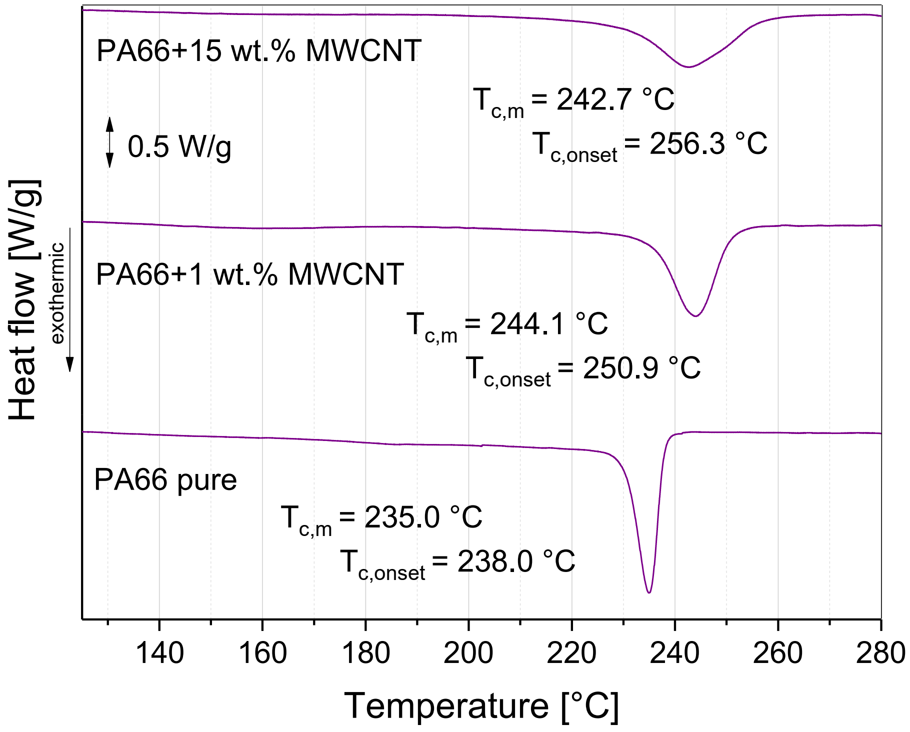

Figure S5. Crystallization behavior of PA66, PA66/1 wt % MWCNT (masterbatch dilution), and PA66/15 wt % MWCNT (masterbatch Plasticyl™ PA1501).

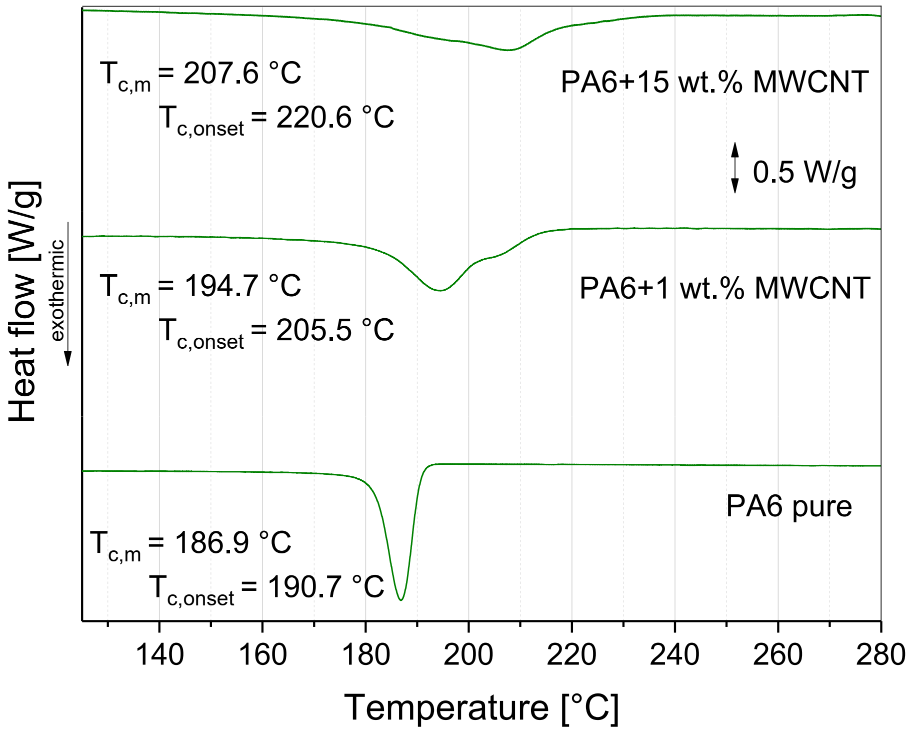

Figure S6. Crystallization behavior of PA6, PA6/1 wt % MWCNT (masterbatch dilution), and PA6/15 wt % MWCNT (masterbatch Plasticyl™ PA1503).

Table S1. Melting temperatures ΔH_m of PA6 and PA66 in PA66/PA6 blends filled with 0 or 1 wt % MWCNT.

Blend composition	ΔH_m [°C] PA6		ΔH_m [°C] PA66	
	@ 0 wt.% CNT	@ 1 wt.% CNT	@ 0 wt.% CNT	@ 1 wt.% CNT
**PA66**	-	-	85.0	83.2
**PA66/PA6 90/10**	-	-	78.4	78.5
**PA66/PA6 80/20**	-	-	75.7	82.4
**PA66/PA6 70/30**	13.2	19.8	53.1	55.2
**PA66/PA6 60/40**	26.3	35.4	44.9	53.8
**PA66/PA6 50/50**	43.6	32.1	37.1	36.3
**PA66/PA6 40/60**	46.5	42.1	30.9	31.7
**PA66/PA6 30/70**	49.8	56.2	14.2	24.1
**PA66/PA6 20/80**	58.7	76.7	18.6	14.4
**PA66/PA6 10/90**	67.4	72.1	7.0	6.2
**PA6**	67.7	80.6	-	-

Rheological measurements

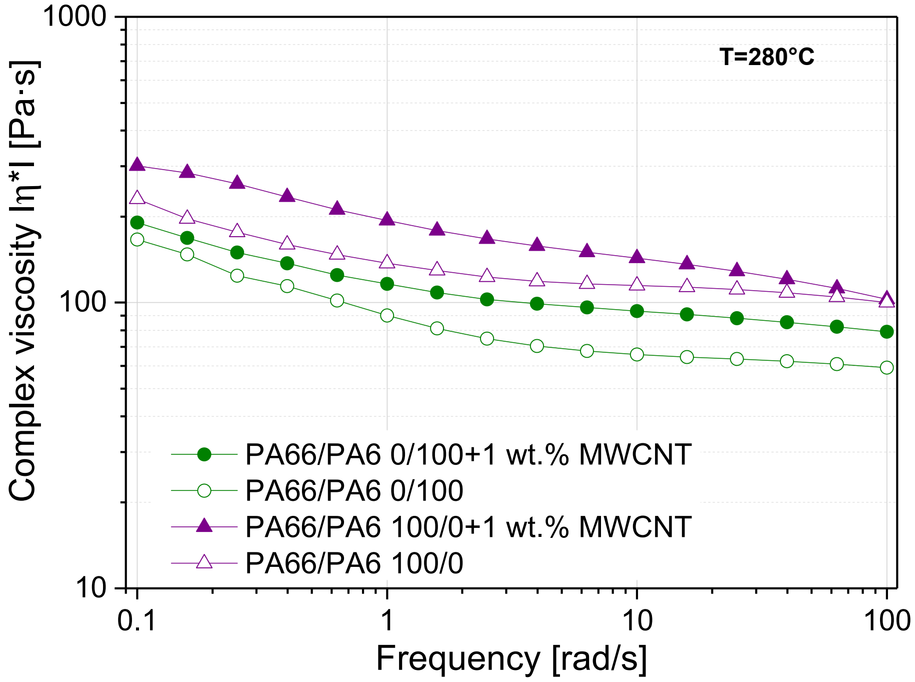

Figure S7. Complex viscosity of PA6 and PA66 and their composites filled with 1 wt % MWCNT.

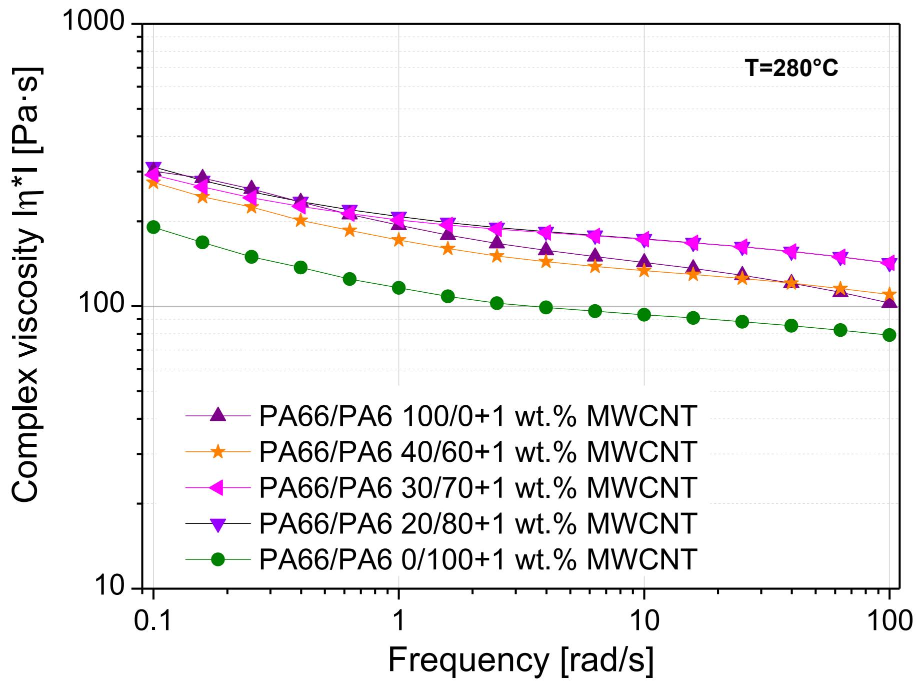

Figure S8. Complex viscosity of PA66/PA6/1 wt % MWCNT blends.
